# Optical coherence tomography with voxel-based morphometry: a new tool to unveil focal retinal neurodegeneration in multiple sclerosis

**DOI:** 10.1093/braincomms/fcad249

**Published:** 2023-09-28

**Authors:** Su-Chun Huang, Marco Pisa, Simone Guerrieri, Gloria Dalla Costa, Giancarlo Comi, Letizia Leocani

**Affiliations:** Experimental Neurophysiology Unit, Institute of Experimental Neurology-INSPE, San Raffaele Scientific Institute, Milan 20132, Italy; Experimental Neurophysiology Unit, Institute of Experimental Neurology-INSPE, San Raffaele Scientific Institute, Milan 20132, Italy; Experimental Neurophysiology Unit, Institute of Experimental Neurology-INSPE, San Raffaele Scientific Institute, Milan 20132, Italy; Experimental Neurophysiology Unit, Institute of Experimental Neurology-INSPE, San Raffaele Scientific Institute, Milan 20132, Italy; Faculty of Medicine, Vita-Salute San Raffaele University, Milan 20132, Italy; Department of Neurorehabilitation Science, Casa di Cura Igea, Milan 20144, Italy; Experimental Neurophysiology Unit, Institute of Experimental Neurology-INSPE, San Raffaele Scientific Institute, Milan 20132, Italy; Faculty of Medicine, Vita-Salute San Raffaele University, Milan 20132, Italy

**Keywords:** multiple sclerosis, biomarkers, optical coherence tomography, multiple sclerosis progression, voxel-based morphometry

## Abstract

Neurodegeneration is the main contributor to disability accumulation in multiple sclerosis. Previous studies in neuro-ophthalmology have revealed that neurodegeneration in multiple sclerosis also affects the neuro-retina. Optical coherence tomography has been used to measure thinning of retinal layers, which correlates with several other markers for axonal/neuronal loss in multiple sclerosis. However, the existing analytical tools have limitations in terms of sensitivity and do not provide topographical information. In this study, we aim to evaluate whether voxel-based morphometry can increase sensitivity in detecting neuroaxonal degeneration in the retina and offer topographical information. A total of 131 people with multiple sclerosis (41 clinically isolated syndrome, 53 relapsing-remitting and 37 progressive multiple sclerosis) and 50 healthy subjects were included. Only eyes with normal global peripapillary retinal nerve fibre layer thickness and no history of optic neuritis were considered. Voxel-based morphometry and voxel-wise statistical comparisons were performed on the following: (i) patients at different disease stages and 2) patients who experienced the first demyelination attack without subclinical optic neuritis, assessed by visual evoked potentials. Standard parameters failed to discern any differences; however, voxel-based morphometry–optical coherence tomography successfully detected focal macular atrophy of retinal nerve fibre layer and ganglion cell/inner plexiform layer, along with thickening of inner nuclear layer in patients who experienced the first demyelination attack (disease duration = 4.2 months). Notably, the atrophy pattern of the ganglion cell/inner plexiform layer was comparable across disease phenotypes. In contrast, the retinal nerve fibre layer atrophy spread from the optic nerve head to the fovea as the disease evolved towards the progressive phase. Furthermore, for patients who experienced the first neurological episode, the severity of retinal nerve fibre layer atrophy at entry could predict a second attack. Our results demonstrate that voxel-based morphometry–optical coherence tomography exhibits greater sensitivity than standard parameters in detecting focal retinal atrophy, even at clinical presentation, in eyes with no history of optic neuritis and with normal latency of visual evoked potentials. Thinning of the ganglion cell/inner plexiform layer primarily concentrated in nasal perifovea in all disease phenotypes, indicating selective vulnerability of retinal ganglion cells and their perifoveal axons. Conversely, the degree of retinal nerve fibre layer thinning seems to be related to the clinical course of multiple sclerosis. The findings suggest bidirectional neurodegeneration in the visual pathway. Voxel-based morphometry–optical coherence tomography shows potential as a valuable tool for monitoring neurodegeneration on a patient level and evaluating the efficacy of novel neuroprotective treatments.

## Teaser

Huang *et al*. applied voxel-based morphometry to enhance sensitivity in detecting neurodegeneration with optical coherence tomography in people with multiple sclerosis. Neuronal and dendritic atrophy was detected after the first demyelinating attack, while baseline axonal atrophy predicted a second attack.

## Graphical Abstract

**Graphical abstract:**
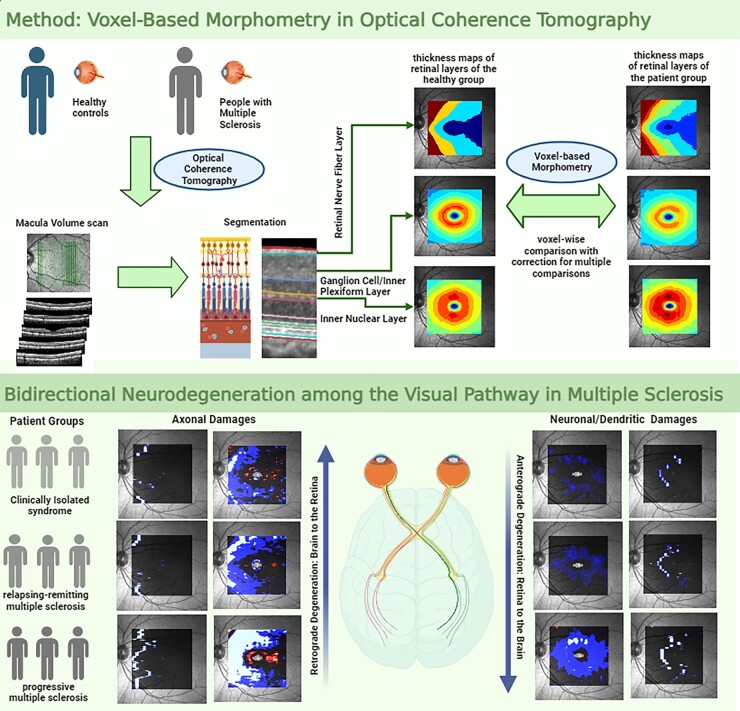

## Introduction

Neurodegeneration is the primary contributor to irreversible disability accumulation in people with multiple sclerosis (PwMS).^1^ Previous studies suggest bidirectional (anterograde and retrograde trans-synaptic) degeneration following the initial axonal/neuronal insults, propagating the neurodegeneration process in multiple sclerosis.^2^ Post-mortem studies showed that neurodegeneration in multiple sclerosis exhibits specific predilections for certain sites and pathways, rather than affecting the entire brain equally, highlighting the importance of investigating how neurodegeneration spreads within the CNS.^3,4^ Such studies are crucial for developing biomarkers to evaluate neuroprotective treatments and also gaining insight onto the pathophysiological mechanisms involved in multiple sclerosis.^5^

The visual pathway is a suitable model for studying neurodegeneration as its well-organized streamlined arrangement.^6^ Recent advances in optical coherence tomography (OCT) enable precise quantification of retinal structures. The thickness of retinal layers containing neuronal cell bodies and dendrites [ganglion cell layer/inner plexiform layer (GCIPL)] or unmyelinated axons (retinal nerve fibre layer [RNFL]) reflects underneath structural loss.^7^ The OCT-derived retinal atrophy has been shown to be a consequence of inflammatory infiltrates along the anterior optic pathway both within and outside the areas of demyelination, or secondary to injury of the posterior visual pathway through retrograde trans-synaptic degeneration.^8-12^ Therefore, the topographical distribution of retinal atrophy in eyes without history of optic neuritis (ON) could be related to subclinical inflammatory activity affecting the optic nerve, or the trans-synaptic neurodegeneration consequent to lesions distributed in the posterior visual pathway, or reveal patterns of selective neuronal vulnerability in multiple sclerosis.^4,13-15^ These different pathophysiological mechanisms may be variably combined in individual patients.

The current OCT analytical tools offer limited topographical information, often reporting global averages for parameters such as peripapillary RNFL thickness (pRNFL) and total macular volume, two of the most used parameters for OCT. This approach reveals only large-scale changes, potentially missing focal abnormalities and reducing the sensitivity of detecting early neurodegeneration in multiple sclerosis, particularly at the patient level.^9^ Therefore, applying neuroimaging tools such as voxel-based morphometry (VBM), which is able to describe the topographical distribution of the OCT-derived retinal thickness at a voxel level, could potentially improve the sensitivity for probing focal neuroaxonal loss *in vivo*. The algorithm for deformable registration of VBM-OCT has been proposed and validated.^16,17^ Hereby, we hypothesize that by implementing VBM to macula OCT images with statistical mapping, we can increase the sensitivity of OCT to consistently detect axonal/neuronal loss at the disease onset of multiple sclerosis and also follow how the neurodegeneration spreads within the visual pathway. To ensure that the alternations in the retinal layers were owing to neurodegeneration associated with multiple sclerosis and not due to acute inflammatory damages, we included only eyes without history of ON and with normal global pRNFL. Additionally, we excluded eyes with an inter-eye difference of pRNFL greater than 5 µm to avoid the presence of subclinical ON.^18^

In the present study, we examined the potential of VBM-OCT to detect neurodegeneration from the onset of multiple sclerosis and follow its trajectory during disease progression. First, we evaluated the voxel-wise reliability of VBM-OCT in healthy controls. Second, we applied this technique to three groups of PwMS representing three phases of the disease trajectory: at diagnosis, during the relapsing-remitting phase (RMS) and those who recently entered the progressive phase (PMS). Despite all subjects having normal global pRNFL and no history of ON, VBM-OCT not only captured alternations in all the retina layers examined but also provided topographical information on how neurodegeneration propagates in the retina. VBM-OCT demonstrated higher sensitivity in capturing retinal atrophy in patients who experienced their first CNS demyelinating attack, in eyes without clinical or subclinical ON [assessed by visual-evoked potential (VEP)]^19^ while standard OCT parameters failed to discern the differences. Further, the severity of RNFL atrophy detected by VBM-OCT at the first entry predicted a second attack. VBM-OCT offers an improved approach for topographic analysis of conventional OCT data, increasing sensitivity in detecting and monitoring neurodegeneration in multiple sclerosis and potentially useful in evaluating the efficacy of novel neuroprotective treatments for individual patient assessment.

## Materials and methods

### Subject

The inclusion criteria for all the participants were the absence of ophthalmological or systemic pathologies that could potentially interfere with visual assessment, such as refractive errors ≤ −6.0 dioptres, macular pathologies, diabetes and optic neuropathies.

Patients attending or referred to the Department of Neurology at the IRCCS San Raffaele Hospital (Milan, Italy) between September 2013 and September 2018 for a neurological episode suggestive of inflammatory CNS disease or routine multiple sclerosis evaluation and treatment were asked to participate in VEP/OCT examinations for research purposes. The inclusion criteria were as follows: (i) a diagnosis of clinically isolated syndrome (CIS) or multiple sclerosis confirmed by the treating neurologists based on 2010 McDonald criteria,^20^ (ii) no history of ON in at least one eye and (iii) Global pRNFL within the normal range according to the manufacturer’s normative database. Additionally, for the patients experiencing their first demyelination attack, normal latency of full-field pattern reversal VEP was an additional inclusion criterion.

Out of 363 patients who underwent the VEP/OCT examinations., we excluded 81 patients with abnormal bilateral pRNFL but no history of ON, 22 patients with history of bilateral ON, 25 patients without macular scans, 34 patients with low-quality scans and 8 patients with bilateral retinopathy. Furthermore, 62 patients at the first CNS demyelination episode were excluded due to abnormal bilateral VEP latency. For the remaining 131 patients, 53 eyes were excluded for ON history (53 eyes), 3 eyes for abnormal pRNFL, 2 eyes for pRNFL asymmetry > 5 µm^18^ and 6 eyes for low-quality images. Some patients/eyes were excluded for more than one reason. Overall, 198 eyes from 131 patients were included in the final analysis **(**Supplementary Fig. 1**).**

A cohort of 50 healthy controls (HC) with similar age and sex distribution and normal pRNFL was also enrolled. The study received approval from the ethical committee of San Raffaele Scientific Institute and was conducted in accordance with the Declaration of Helsinki. Written informed consent was obtained from all study participants.

### Clinical data

For each patient, age, sex, date of first symptom onset and Expanded Disability Status Scale (EDSS) score were evaluated by the treating neurologists. All patients underwent VEPs as part of the inclusion criteria. VEPs were recorded in response to reversal achromatic checks with stimulation sizes of 60, 30 and 15 min of arc of the visual angle over midline occipital (Oz) area, with midline central (Cz) as the reference. Refractive corrections were used during VEP recording when necessary. VEP latencies were compared with normative data obtained in our department, as previously published.^19^ A latency which exceeded 2.5 standard deviations from the normative data for any stimulation size (60’, 30’ and 15’) was considered as delayed/abnormal VEP.

### OCT acquisition protocol

Retinal imaging was acquired with a spectral-domain OCT scanner (Spectralis, Heidelberg Engineering, Heidelberg, Germany). The OCT scanning and VEP recording were performed on the same day by two experienced neurologists (M.P. and S.G.). Bilateral peripapillary and macular volume scans were acquired on each subject with the automatic real-time (ART) mode activated. The peripapillary scans were centred at the optic nerve head (ONH) with a 12° ring manually placed. The scan was performed in high-resolution mode (1536 A-scans) with the depth resolution of 3.87 µm, and the ART was set at 100. The macula volume scans centred at the fovea with a coverage of 20 × 20 degrees. The volume scan consisted of 25 vertical B scans, and each B scan was composed of 1024 A-scans in high-resolution mode. The distance between slices is 240 µm, and the ART ranging from 15 to 25 were used. The quality assessment was performed by S.C.H., and only images with quality scores higher than 25 and meeting the OSCAR-IB criteria for OCT analysis were used.^21^

To assess the reliability of the VBM-OCT analysis, 10 healthy subjects underwent a repeated OCT scan on the same day using the TruTrack active eye tracking mode.

The images were automatically segmented by the Heidelberg Eye Explorer (Heyex, version 1.10.2.0). The segmented results were visually inspected, and manual corrections were made if necessary. The raw images and the segmentation results were exported for further VBM analysis. Standard OCT parameters, including pRNFL, thickness of temporal sector of pRNFL (tRNFL), macula RNFL volume (mRNFL), macula GCIPL volume (mGCIPL) and macula inner nuclear layer volume (mINL), were also generated from the Heyex software for further analysis. In the case of a subject that has OCT scans of both eyes, the data were averaged. The OCT data were reported in alignment with the recently updated APOSTEL 2.0 recommendations.^22^

### VBM pipeline

The registration algorithm employed for the VBM analysis was based on the A-OCT registration proposed by Chen *et al*.,^16^ which consists of a 2D global translation of the whole volumetric scan and a series of 1D affine transformations of each A-line. The transformation matrices obtained from these two steps were then applied to the segmented maps, to register them to the target space and enable voxel-wise comparison.

The registration was performed with self-developed scripts in MATLAB R2016b (MathWorks, Natick, MA, USA). First, the whole macula volumetric scans were flipped into the orientation of the left eyes, with the ONH appearing on the left side. Subsequently, intensity normalization was applied to ensure similar intensity levels among the images. Next, the retina area, spanning from the internal limiting membrane (ILM) to Bruch’s membrane (BM), was extracted. The fovea was identified as the thinnest point of the retina, serving as a landmark for 2D global translation. The global translation linearly registered the normalized images of each individual to the target space by aligning the fovea. Following this, a 1D affine transformation was performed on each A-scan to match the subject’s retina to the target. The transformation matrices obtained from the aforementioned steps were applied to the segmented maps exported directly from the Heyex software, namely, RNFL, GCIPL, inner nuclear layer (INL) and Retina (i.e. retinal thickness—ILM to BM).

Upon completion of the VBM pipeline, four retinal maps (RNFL, GCIPL, INL and Retina maps) were generated of each group. The central fovea area was excluded during voxel-wise statistical analyses.

### Statistical analysis

**Figure 1:**
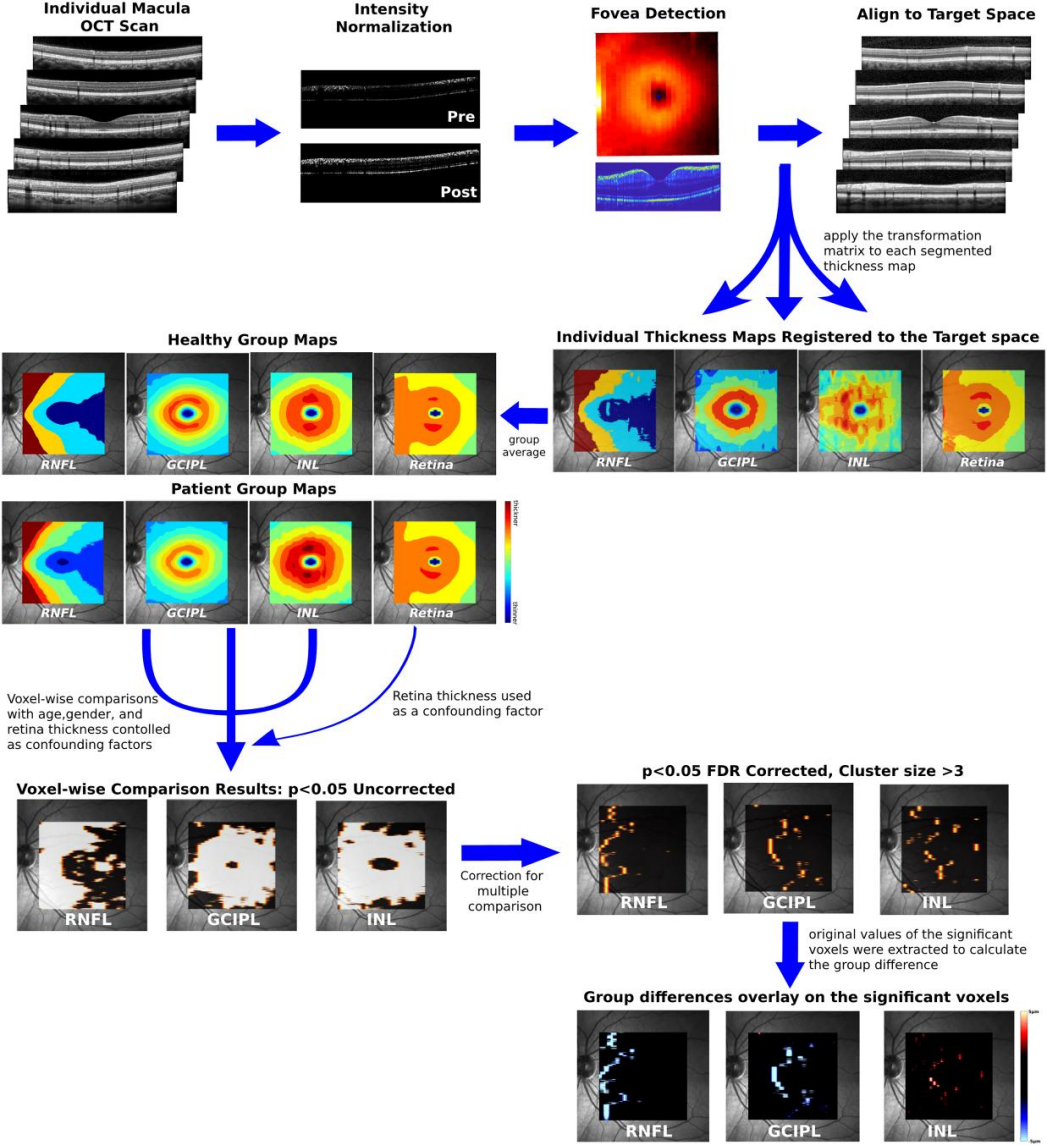
**Analytical flowchart of VBM-OCT pipeline.** The top row illustrates the spatial registration procedure of individual OCT scans to the target space, ensuring alignment for further analysis. The second row depicts the creation of group maps, enabling the comparison between different groups. The third row shows raw results of voxel-wise comparisons and the correction for multiple comparisons. The false discovery rate (FDR) was controlled, and cluster-based analysis with a minimum cluster size of 3 was applied to enhance statistical robustness. To visually interpret the findings, the thickness differences of the significant voxels were extracted from each individual map and superimposed onto the fundus image in the last row.

Voxel-wise comparisons at the group level were performed with a general linear model (GLM).^23^ Univariate GLM was used to conduct voxel-wise comparisons on RNFL, GCIPL and INL maps, respectively, while the Retina maps were used as covariates. To address errors arising from multiple comparisons, false discovery rate (FDR) correction and cluster-based analysis were applied.^24,25^ Voxels with *P*-values less than the alpha level (set at 0.05) after FDR correction and a cluster size greater than 3 (9 adjacent voxels) were considered statistically significant. For visualization of the results, voxels that showed significant differences were superimposed on the fundus image, with colours representing the thickness difference with respect to the HCs (disease group—HC group). The VBM pipeline and GLM procedure were summarized in Fig. 1.

For each map (RNFL, GCIPL and INL), a percentage change map was calculated as follows:


(1) $$\mathrm{Change}\;(\text{\%})=\frac{\left(\mathrm{patient}\;\mathrm{map}-\mathrm{HC}\;\mathrm{map}\right)}{\mathrm{HC}\;\mathrm{map}}\;\times 100.$$

Voxel-wise comparisons were performed with age, gender and retina thickness as covariates in two contexts: (i) among the HC group and the PwMS groups and (ii) between the HC group and patients who experienced their first demyelination event. To further examine the effects of confounding factors on the final thickness measurements, a voxel-wise regression analysis was conducted using GLM to evaluate the effects of age, disease duration and EDSS on the RNFL, GCIPL and INL thickness maps.

Voxel-wise reliability was evaluated by calculating the within-subject standard deviation (Sw), coefficient of variance (CV) and interclass correlation coefficient (ICC) of each voxel.

## Results

### Subject demographics

One hundred and thirty-one PwMS or those who experienced a first CNS demyelinating event suggestive of multiple sclerosis were included in the analysis. The patients were divided into three groups based on their diagnosis, including CIS (*n* = 41), relapsing-remitting multiple sclerosis (RMS, *n* = 53), and progressive multiple sclerosis (PMS, *n* = 37, including 17 primary and 20 secondary PMS). Among the entire cohort, 50 patients were experiencing their first CNS demyelinating episode, which could not be attributed to other diseases. The OCT acquisition time for this cohort was performed at 0.4 ± 0.3 years after the first attack (ranging from 0.01 to 1 year, and no patient experienced a second attack during this period). At the time of the OCT assessment, none of the patients were under disease-modifying therapies (DMT). Among them, nine were diagnosed with clinical definite multiple sclerosis (CDMS) according to the diagnostic criteria in use at the time of discharge (Polman, 2011); for the others, a diagnosis of CIS was made after ruling out alternative diagnoses. Over their routine follow-up period (*average duration* 6.3 ± 1.3 years), 25 individuals were converted to a diagnosis of multiple sclerosis (CIS/CDMS) based on the 2017-revised diagnostic criteria, while 16 of them retained a diagnosis of CIS.^26^ The demographics of study subjects are presented in Table 1.

**Table 1:** Subject demographics

	Whole dataset			
	HC, *n* = 50 (100 eyes)	CIS, *n* = 41 (57 eyes)	RMS, *n* = 53 (88 eyes)	PMS, *n* = 37 (53 eyes)
Gender (*N*, % male)	16, 32.0%	15, 36.6%	21, 39.6%	21, 56.8%‡
Age (y)	34.4 ± 10.0	31.5 ± 8.7	36.2 ± 10.8	46.6 ± 9.1*
Disease duration (y)	-	0.4 ± 0.4	5.5 ± 3.9	8.8 ± 8.6
Progression duration (y)	-	-	-	2.2 ± 1.3
EDSS	-	0.9 ± 0.7	1.3 ± 0.7	5.0 ± 1.1
DMT types (*N*, %)				
High effective	-	0, 0.0%	1, 1.9%	3, 8.1%
Moderately effective	-	5, 12.2%	24, 45.2%	11, 29.7%
Modestly effective	-	2, 4.9%	17, 32.1%	6, 16.2%
No treatment	-	34, 82.9%	11, 20.8%	17, 45.9%
Delayed VEP (%)	-	0%	28.4%	70.0%
pRNFL (µm)	98.8 ± 8.8	97.2 ± 7.4	94.4 ± 8.4	93.4 ± 8.9
tRNFL (µm)	72.3 ± 8.5	69.8 ± 11.8	66.0 ± 9.1*	63.2 ± 12.5*
PMB (µm)	55.0 ± 7.1	53.8 ± 8.4	51.3 ± 6.9	47.7 ± 9.1*
mRNFL (mm^3^)	0.99 ± 0.11	0.95 ± 0.08	0.92 ± 0.11*	0.85 ± 0.11*
mGCIPL (mm^3^)	2.03 ± 0.13	1.95 ± 0.14	1.93 ± 0.14*	1.80 ± 0.19*
mINL (mm^3^)	1.02 ± 0.08	1.05 ± 0.07	1.00 ± 0.08	1.02 ± 0.08

	Patients who experienced the first demyelination attack			
	First attack, *n* = 50 (73 eyes)	CIS/CIS, *n* = 16 (18 eyes)	CIS/CDMS, *n* = 25 (39 eyes)	CDMS, *n* = 9 (16 eyes)
Gender (*N*, % male)	19, 38.0%	7, 43.8%	8, 32%	4, 44.4%
Age (y)	30.7 ± 8.7	30.5 ± 7.2	32.1 ± 9.5	27.1 ± 8.4
Disease duration (y)	0.4 ± 0.3	0.4 ± 0.4	0.4 ± 0.3	0.3 ± 0.2
EDSS	0.9 ± 0.7	0.8 ± 0.7	0.9 ± 0.7	1.0 ± 0.8
ON as entry sign (%)	54%	87.5%	44%	22%
DMT at final follow-up (*N*, %)				
High effective	3, 6.0%	0, 0.0%	3, 12.0%	0, 0.0%
Moderately effective	17, 34.0%	0, 0.0%	9, 36.0%	8, 88.9%
Modestly effective	11, 21.6%	0, 0.0%	10, 40.0%	1, 11.1%
No treatment	19, 38.0%	16, 100%	3, 12.0%	0, 0.0%
pRNFL (µm)	96.6 ± 7.3	97.9 ± 7.7	96.8 ± 7.3	93.8 ± 6.6
tRNFL (µm)	69.4 ± 11.4	73.4 ± 14.6	67.5 ± 9.2	67.7 ± 9.9
PMB (µm)	53.7 ± 8.4	56.7 ± 9.2	52.0 ± 7.4	53.3 ± 8.9
mRNFL (mm^3^)	0.94 ± 0.09	0.99 ± 0.08	0.93 ± 0.07	0.91 ± 0.11
mGCIPL (mm^3^)	1.94 ± 0.14†	2.01 ± 0.14	1.92 ± 0.14†	1.90 ± 0.14
mINL (mm^3^)	1.06 ± 0.07	1.07 ± 0.05	1.05 ± 0.07	1.10 ± 0.07

HC, healthy controls; CIS, clinically isolated syndrome; RMS, relapsing-remitting multiple sclerosis; PMS, progressive multiple sclerosis; EDSS, expanded disability status scale; DMT, disease-modifying therapy; VEP, visual-evoked potential; pRNFL, thickness of global peripapillary retinal nerve fibre layer thickness; tRNFL, thickness of the temporal sector of pRNFL; PMB, papillomacular bundle; mRNFL, volume of macula RNFL; mGCIPL, volume of macula ganglion cell layer/inner plexiform layer; mINL, volume of macula inner nuclear layer; CDMS, clinically definite multiple sclerosis; ON, optic neuritis; -, data not available.

^*^ *P* < 0.01: post-hoc analysis of one-way ANOVA test with the HC group.

^†^ *P* < 0.05: Independent *t*-test with the HC group.

^‡^ *P* < 0.05: χ² test with the HC group.

### Test–retest reliability of VBM-OCT

High test–retest reliability of VBM-OCT was observed in healthy controls, particularly in the nasal macula. Firstly, we calculated the reliability of the standard macula OCT parameters: mRNFL, mGCIPL and mINL. The *CV* and *ICC* values were 1.64% and 0.98 for mRNFL, 0.28% and 0.99 for mGCIPL and 1.5% and 0.99 for mINL, which were consistent with the literature.^27^

**Figure 2:**
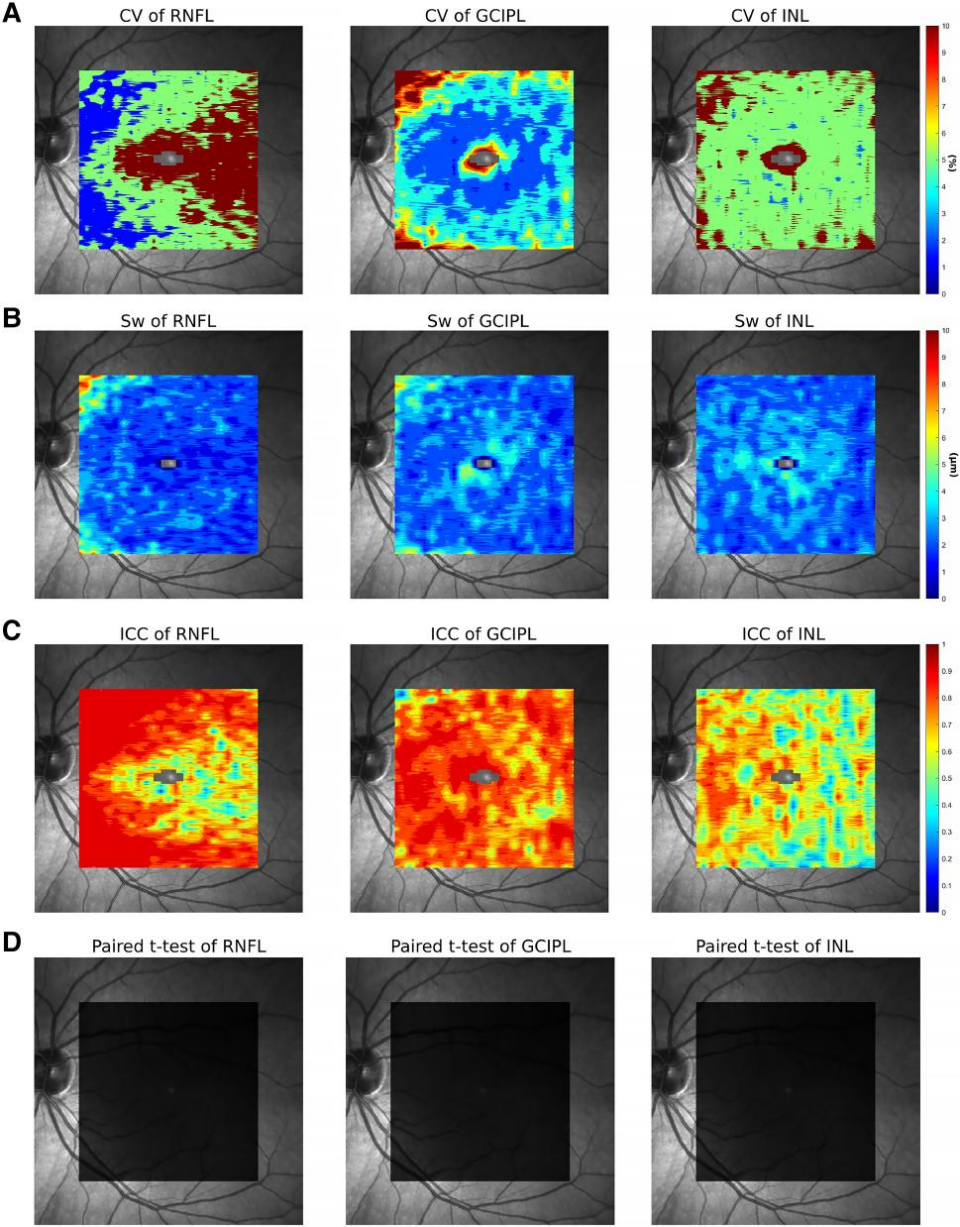
**Voxel-wise reliability.** (**A**) coefficient of variance (CV), (**B**) within-subject standard deviation (Sw) and (**C**) interclass correlation coefficient (ICC) were calculated voxel-wise to evaluate the reliability of RNFL, GCIPL and INL. (**D**) Voxel-wise paired *t*-test results between the 1st and 2nd scans of healthy subjects were analysed to examine the efficacy of correction for multiple comparisons; no voxels showed significant difference after the correction.

Second, we assessed the voxel-wise *Sw*, *CV* and *ICC*  **(**Fig. 2**)**. The *Sw* was consistently low across the whole macula in most voxels (less than 3 µm), while the *CV* and *ICC* demonstrated non-uniform distribution across the macula. For RNFL, test–retest reliability was highest near the optic nerve head (*CV* < 2% and *ICC* > 0.9), gradually reducing towards the temporal macula. Regarding GCIPL, the *CV* was lowest around the peri-foveal zone (<3%), with most of the macula showing good reliability (*ICC* > 0.8). For INL, only the nasal macula reached an *ICC* greater than 0.7. The average *CV* and *ICC* of the whole maps were 8.84% and 0.8260 for RNFL, 4.9% and 0.8267 for GCIPL, 8.33% and 0.6743 for INL and 0.81% and 0.92 for the retina, respectively. Good reliability (*ICC* > 0.75) was found in RNFL and GCIPL maps, while the INL showed moderate reliability.^28^

Lastly, a voxel-wise paired *t*-test was performed between the 1st and 2nd scans of the HCs to examine the efficacy of the correction for multiple comparisons (Fig. 2D). After the correction, no voxel showed significant difference.

### Patterns of retinal abnormality at different disease stages

We initially applied VBM-OCT to PwMS at three representative phases of the disease trajectory: CIS, relapsing-remitting, and progressive phase. Only eyes without clinical ON and with normal global pRNFL were included in the analysis. The sensitivity of VBM-OCT was compared to other standard OCT parameters (*tRNFL*, *mRNFL*, *mGCIPL* and *mINL*).

For axonal damages, standard OCT detected *tRNFL* and *mRNFL* atrophy in the RMS and PMS groups compared with HCs (RMS: 8.71% and 7.07%; PMS: 12.59% and 14.14%), while no difference was found in the CIS group compared with HCs. In contrast, VBM-OCT detected RNFL thinning in all groups (6.43% for CIS, 7.84% for RMS and 14.80% for PMS). Concerning neuronal loss, standard parameter *mGCIPL* detected atrophy only in the RMS and PMS groups (4.93% and 11.33%, respectively), while no difference was found in the CIS group. VBM-OCT, on the other hand, detected GCIPL atrophy in all groups (4.81% for CIS, 5.25% for RMS and 8.59% for PMS). No difference of INL was detected with standard parameters, whereas VBM-OCT detected INL thickening in all patients (11.04% for CIS, 12.14% for RMS and 9.63% for PMS).

**Figure 3:**
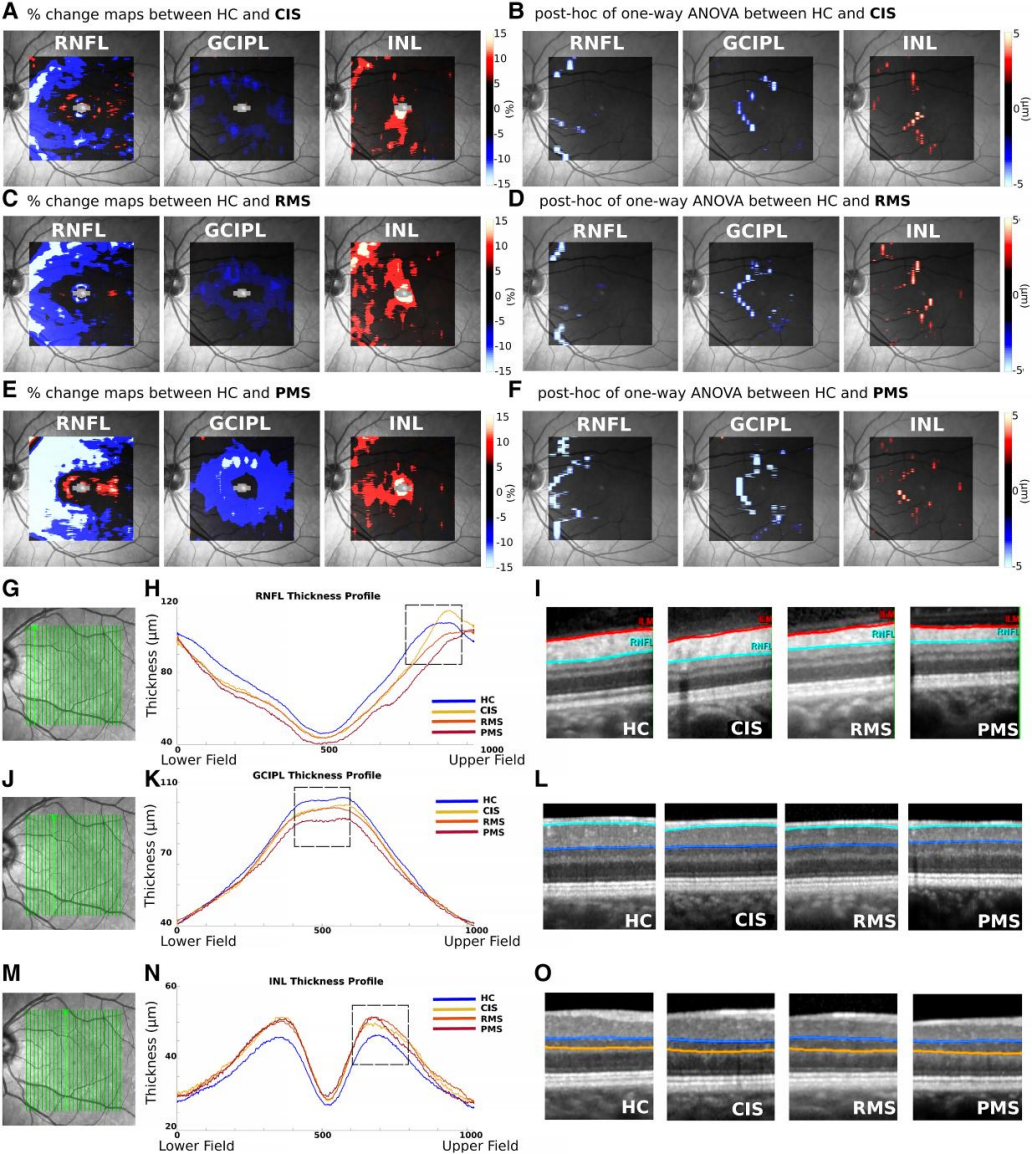
**VBM-OCT results between the HC and the PwMS groups.** Percentage change maps of the RNFL, GCIPL and INL between CIS (**A**), RMS (**C**) and PMS (**E**) with the HC groups are depicted (colour bar ranges from ± 15%). Voxel-wise ANOVA results of RNFL, GCIPL and INL between CIS (**B**), RMS (**D**) and PMS (F**)** with the HC groups are shown (colour bar ranges from ± 5 µm). Lower part of the figure shows representative OCT images for each group. The locations of the OCT scans are indicated in the fundus images (**G**, **J**, **M**). The thickness profiles of RNFL (**H**), GCIPL (**K**) and INL (**N**) for each group are illustrated. Focal OCT images within the marked box on the thickness profile are shown in (**I**) for RNFL, between the red and turquoise lines; (**L**) for GCIPL, between the turquoise and blue lines; (**O**) for INL, between the blue and orange lines.

The percentage change maps of the results of voxel-wise comparisons between the disease groups and HCs are shown in Fig. 3. VBM-OCT revealed significant RNFL and GCIPL atrophy, as well as INL thickening in all disease groups.

The degree of RNFL atrophy gradually increased as the disease moved towards to the progressive phase (−3.54 µm for CIS, −5.38 µm for RMS and −10.14 µm for PMS). From a topographical standpoint, the RNFL atrophy is observed in proximity to the ONH in the CIS group (Fig. 3A). The extent of atrophy worsens in the RMS group, involving a larger area that encompasses regions near to the ONH as well as the superior and inferior perifoveal zones (Fig. 3C). In the PMS group, atrophy occupies the entire temporal macula, spanning the region between the ONH and the fovea **(**Fig. 3E**).**

Significant perifoveal GCIPL atrophy was observed with VBM-OCT, predominantly located in the perifoveal zone, especially in the nasal macula. GCIPL atrophy also increased with disease severity, with lower level observed in the CIS and RMS groups (−4.23 µm for CIS and −4.64 µm for RMS) compared to the PMS group (−7.11 µm).

The presence of INL thickening mirrored GCIPL thinning. The averaged INL thickening was 3.86 µm for CIS, 4.36 µm for RMS and 3.51 µm for PMS.

### Topographical association of RNFL, GCIPL and INL changes at different disease stages

**Figure 4:**
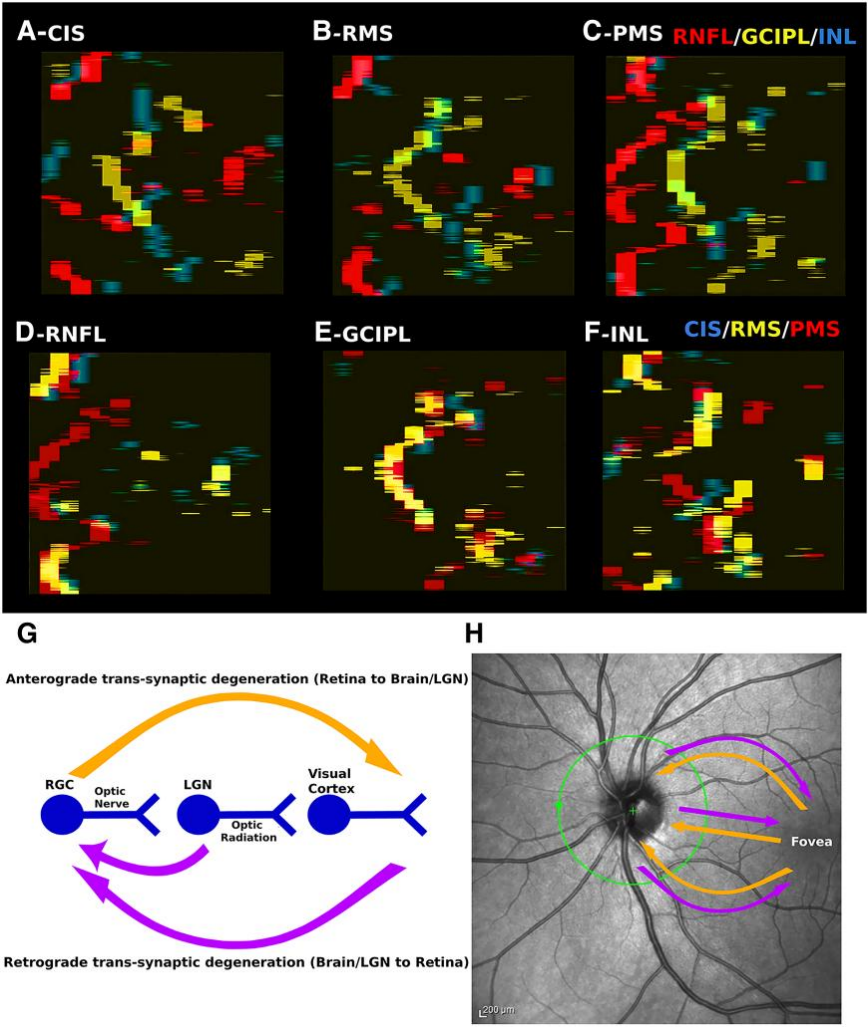
**Retinal layer alternations and bidirectional neurodegeneration.** Intra-group comparison maps showing significant changes in RNFL (red), GCIPL (yellow) and INL maps (blue) between the HC group and CIS (**A**), RMS (**B**) and PMS (**C**) groups, respectively. Inter-group comparison maps illustrating significant changes in the CIS (blue), RMS (yellow) and PMS (red) groups of RNFL (**D**), GCIPL (**E**) and INL (**F**). Based on the consistent pattern between the observed RNFL atrophy and the fibre projections, we speculate that the pattern of RNFL atrophy suggests retrograde degeneration from the axons of the RGCs towards their cell bodies. In alignment with this hypothesis, GCIPL atrophy is limited to the nasal perifoveal zones, as indicated by the yellow areas in (**A**), (**B**) and (**C**). Notably, this location remains consistent across all three subgroups of multiple sclerosis (Fig. 4E). The discrepancy between RNFL and GCIPL atrophy patterns suggests that GCIPL atrophy is not solely due to retrograde degeneration from axonal damage but rather involves primary neurodegeneration targeting the soma of RGCs. (**G**) Bidirectional neurodegeneration can be captured in the retina with VBM-OCT. GCIPL atrophy may lead to anterograde degeneration towards the brain (orange arrow), while RNFL atrophy may be related to retrograde degeneration from the brain to the retina (purple arrow). (**H**) The bidirectional degeneration in the retina follows the direction of the fibre projection of RGCs (orange arrows), resulting in pronounced atrophy in the temporal RNFL. LGN, lateral geniculate nucleus.

The VBM-OCT *t*-test results from Fig. 3 were overlapped to further explore the topographical relationship between changes in different retinal layers within and between the PwMS subgroups. The significant voxels of RNFL, GCIPL and INL were superimposed for the CIS (Fig. 4A), RMS (Fig. 4B) and PMS (Fig. 4C) groups. Concomitant GCIPL thinning and INL thickening, along with RNFL thinning concentrated around the ONH, and were observed in all groups.

Furthermore, we overlapped the results based on the layers, superimposing the significant voxels of RNFL for CIS, RMS and PMS in Fig. 4D, GCIPL in Fig. 4E and INL in Fig. 4F. Interestingly, changes in retinal layers exhibited similar patterns across disease stages: RNFL atrophy accumulated around the ONH, GCIPL atrophy condensed in the nasal perifoveal zone and INL thickening was prominent in the parafoveal zone.

### Sensitivity of VBM-OCT in patients who experienced the first demyelination attack

**Figure 5:**
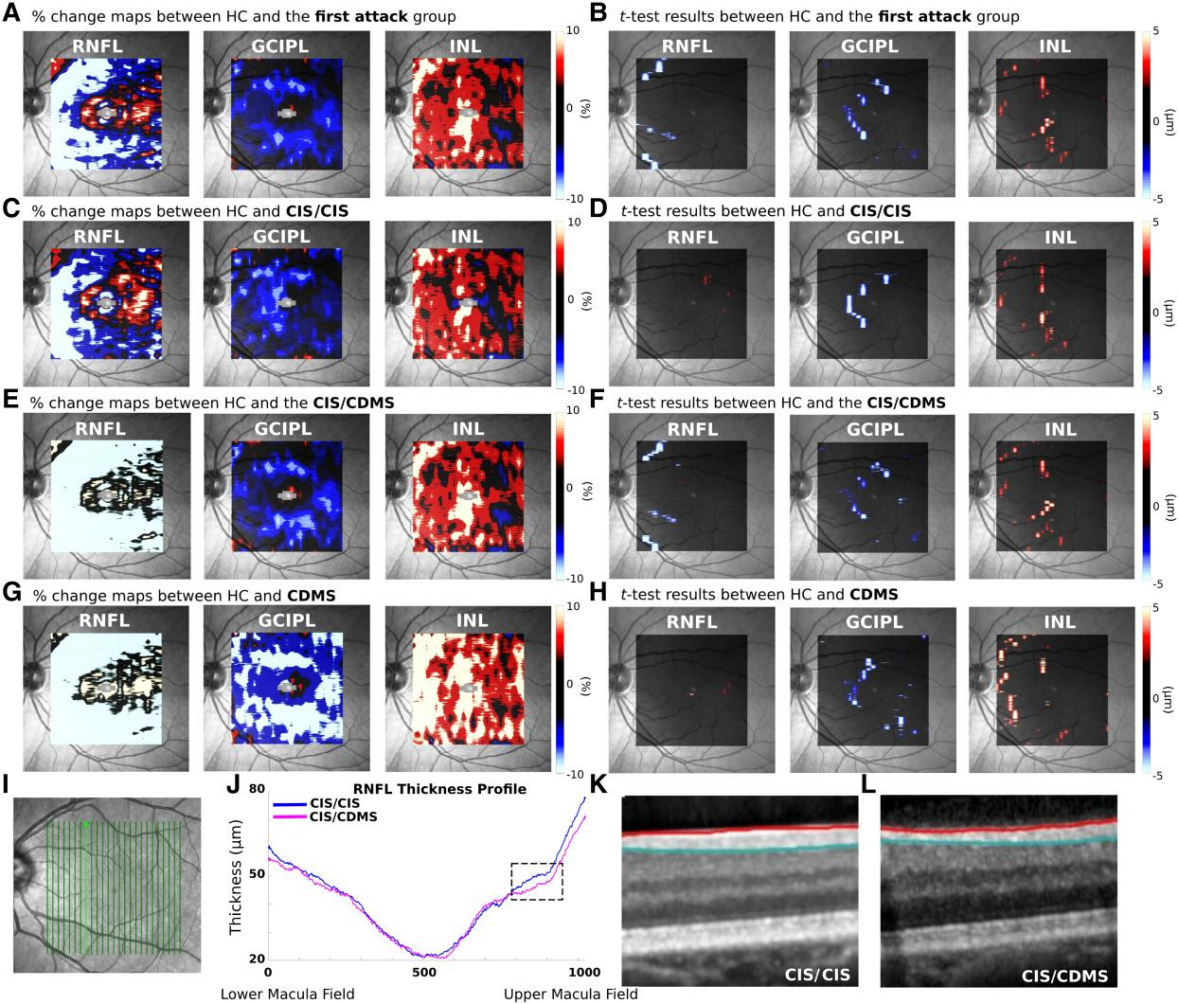
**VBM-OCT results between the HC group and patients who experienced their first demyelinating attack.** The left column shows the percentage change map (colour bar ranges from ± 10%) and the right column displays the voxel-wise *t*-test results (colour bar ranges from ± 5 µm). (**A**, **B**) Comparison between the HC group and the entire patient group who experienced their first demyelinating attack. (**C**, **D**) Comparison between the HC group and the CIS/CIS. (**E**, **F**) Comparison between the HC group and the CIS/CDMS. (**G**, **H**) Comparison between the HC group and the CDMS. (**I**) Fundus image indicating the scanning location of OCT sample images. (**J**) RNFL thickness profile of the slides shown in **I**. (**K**) OCT images of the black box for CIS/CIS. (**L**) OCT images of the black box for CIS/CDMS.

We included a group of patients who experienced their first CNS demyelination event, using only eyes without clinical or subclinical optic nerve involvement (i.e. normal VEP conduction, normal and no asymmetry of global pRNFL, as previously published).^19^ Among standard OCT parameters, only *mGCIPL* showed 4.43% atrophy compared to the HCs, while no other significant difference was found. Instead, VBM-OCT detected significant RNFL (−3.85 µm, 7.04%) and GCIPL atrophy (−4.22 µm, 4.96%), as well as INL thickening (4.08 µm, 11.76%) compared with the HC group (Fig. 5A and B).

We further separated the cohort based on their diagnosis at hospital discharge and at routine follow-ups (6.3 ± 1.3 years). For patients who remained diagnosed with CIS at follow-ups (CIS/CIS), no difference in standard OCT parameters was found, while VBM-OCT showed GCIPL atrophy (−6.04 µm, 6.22%) and INL thickening (4.16 µm, 11.72%) compared to HC, but no significant RNFL atrophy was found (Fig. 5C and D). In the group that converted to clinically definite MS (CIS/CDMS), only one standard parameter, *mGCIPL*, showed a reduction (−0.12 mm^3^, 5.91%). However, VBM-OCT detected GCIPL atrophy (−3.76 µm, 4.45%), INL thickening (4.27 µm, 12.5%) and RNFL atrophy (−4.75 µm, 8.32%) (Fig. 5E and F). For patients diagnosed with MS at their first neurological episode (CDMS), no significant difference in standard parameters was found compared to the healthy group. Nevertheless, VBM-OCT detected RNFL thickening in a small area in the temporal macula (2.59 µm, 18.84%) and GCIPL thinning (−4.96 µm, 6.00%) and INL thickening (5.79 µm, 18.30%) in the nasal macula (Fig. 5G and H).

When comparing CIS/CIS, CIS/CDMS and CDMS groups, no difference was found in either standard parameters or VBM-OCT results.

Finally, to verify the VBM-OCT results, especially the nasal RNFL atrophy in the CIS/CDMS group, representative OCT images from CIS/CIS and CIS/CDMS are shown (Fig. 5K, L**)**. RNFL atrophy of the CIS/CDMS can be observed in the original OCT images directly.

### Associations between VBM-OCT measures and demographic variables

**Figure 6:**
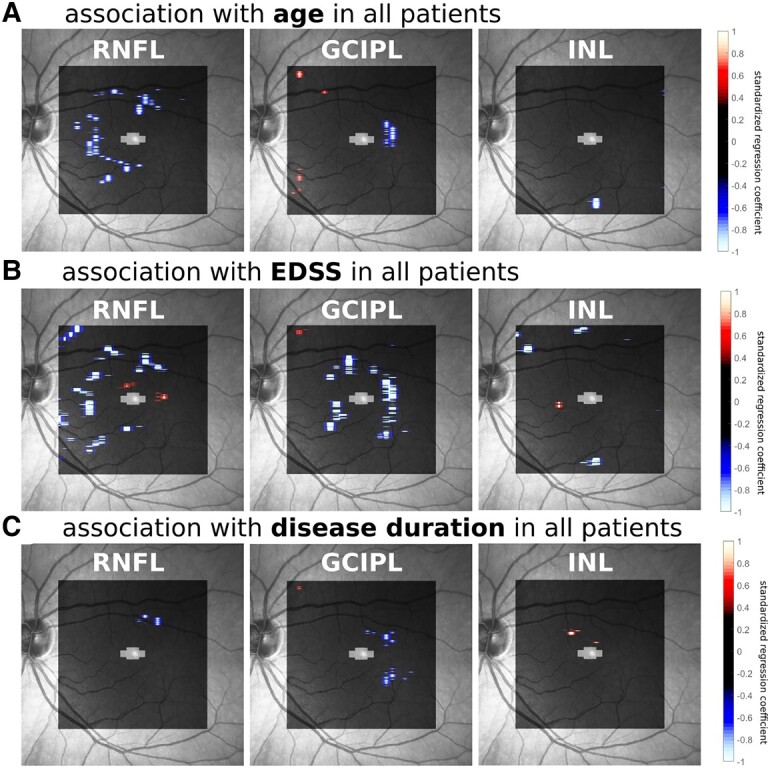
**Effects of confounding factors on the thickness of retinal layers.** Voxels showing significant effects in the linear regression model are illustrated: (**A**) age, (**B**) EDSS scores and (**C**) disease duration. The colour bar represents the standardized regression coefficients (SRC) of the factors.

Linear regression analysis was conducted with GLM to evaluate the association between confounding factors and OCT-measured retinal thickness (Fig. 6). Age and EDSS were negatively correlated with the RNFL thickness in the nasal macula [*standardized regression coefficient* (*SRC*) = −1.04 for age and −1.01 for EDSS], while disease duration negatively correlated with RNFL thickness in the superior temporal macula (*SRC* = −0.94). For GCIPL, age and disease duration were correlated with atrophy in the temporal perifoveal area (*SRC* = −0.91 and −0.85, respectively), and EDSS correlated with GCIPL atrophy around the perifoveal zone (*SRC* = −1.13). For INL, no clear pattern of correlation was found.

## Discussion

Neurodegeneration occurs early in the disease course of multiple sclerosis and is the main determinant of disability. However, a quick, economical and sensitive biomarker that can capture early neuro-axonal loss on the patient level is still lacking. In the present study, we developed and validated an analytical pipeline that enabled us to deliver statistical mapping with VBM to macular OCT in both PwMS and healthy controls. First, we examined test–retest reliability of VBM-derived retinal thickness maps in healthy controls, which demonstrated high reliability. Second, we compared the VBM-OCT maps between healthy controls and PwMS with different disease stages, including CIS, relapsing-remitting (RMS) and those recently entered the progressive phase (PMS). Our data revealed that GCIPL atrophy is an early feature in multiple sclerosis, preferentially affecting the perifoveal zone. This finding suggests a selective vulnerability of RGCs or their perifoveal axons to multiple sclerosis pathogenesis. Additionally, we observed that RNFL atrophy spreads from the ONH towards the fovea as the disease progresses, possibly reflecting retrograde degeneration due to increased disease burden. The discrepancy of RNFL and GCIPL atrophy patterns implies a bidirectional (trans-synaptic) neurodegeneration of the visual pathway. These two mechanisms together explain the preferential vulnerability of temporal RNFL, known as the most sensitive metric to detect neurodegenerative changes in eyes using standard OCT techniques.^29^ Furthermore, our VBM-OCT analysis revealed a spatial association between GCIPL atrophy and INL thickening, supporting the hypothesis that INL thickening may be a reactive response to RGC damage.^19^ Lastly, we conducted voxel-wise comparisons between the HCs and patients who experienced their first demyelination attack, including only eyes without clinical or subclinical optic nerve involvement (assessed by patient history, VEP, global pRNFL and the asymmetry of global pRNFL). Here, VBM-OCT exhibited higher sensitivity compared to standard OCT parameters. RNFL and GCIPL atrophy, as well as INL thickening, were detected with VBM-OCT at the earliest stage of the disease when standard parameters failed to do so. Notably, RNFL atrophy at entry in patients with the first episode predicted a second attack, highlighting the potential prognostic value of VBM-OCT in multiple sclerosis.

### Reliability of the VBM technique in healthy controls

Firstly, we evaluated the reliability of the VBM technique in healthy controls. When using standard parameters (*mRNFL*, *mGCIPL* and *mINL*), excellent reliability was found in all three layers (*ICC* > 0.98 for all), consistent with prior literature.^27^ Next, we performed voxel-wise analyses in the same dataset and observed variability in reliability across the macula, which was related to the absolute thickness of the layers. For example, RNFL thickness was highest near the ONH and gradually decreased towards the temporal macula. As a result, *CV* and *ICC* values exhibited lower variability and higher reliability closer to the ONH. The reliability of GCIPL was consistently good to excellent (*ICC* > 0.75) across the entire macula. However, the INL showed higher variability (*CV* = 8.33%) and moderate reliability compared to the other two layers. Our findings align with prior multicentre reliability test of semiautomatic retinal layer segmentation, where the authors reported lower inter-rater ICC values in the temporal RNFL and INL, and suggested that segmentation accuracy might decrease when absolute thickness is low.^30^ Overall, our results indicate that VBM-OCT demonstrates higher reliability in the nasal macula (between ONH and the fovea), especially for RNFL and INL, while changes in the temporal macula may be underestimated.

### VBM-OCT improves sensitivity in detecting retinal changes in multiple sclerosis

It has been well documented that *pRNFL* and *mGCIPL* can be used as markers to monitor neurodegeneration in multiple sclerosis.^31-34^ Thinning of *pRNFL*, atrophy of *mGCIPL* and reduction of *TMV* have been reported in both CIS and various multiple sclerosis subtypes, and these retinal changes were correlated with clinical and cognitive deterioration as well as brain atrophy.^34-40^ However, most of these findings were demonstrated in a more advanced phase of the disease, rather than at initial diagnosis, and often involved larger sample sizes. With the aid of VBM-OCT, we observed significant atrophy of RNFL and GCIPL, as well as INL thickening, at the earliest stages of the disease (i.e. CIS patients at clinical onset with a mean disease duration of 4.8 months). Notably, these changes were detected in eyes without any clinical manifestations and no evidence of subclinical damage, as demonstrated by normal VEPs. These results are consistent with recent work from Pietroboni *et al*.,^41^ which reported atrophy of mRNFL and mGCIPL in early RMS (*mean disease duration* = 23 months).

Moreover, our results revealed that patients diagnosed with multiple sclerosis at the first attack or during follow-up exhibited more pronounced RNFL atrophy compared to those initially diagnosed with CIS who maintained a CIS diagnosis throughout the follow-up period (Fig. 5). Despite variations in the time lapse between the attack and the OCT acquisition within the cohort, there were no significant differences in the time to OCT acquisition among the three groups (CIS/CIS, CIS/CDMS and CDMS). Additionally, we conducted an analysis excluding those with a time to OCT acquisition of more than 6 months (*N* = 7), and the results remained consistent.

### VBM-OCT maps show specific and distinct patterns of RNFL and GCIPL thinning at different stages of multiple sclerosis

The VBM-OCT results at different disease stages showed that as the disease progresses, RNFL thinning not only increases in severity (as the level of atrophy showed a linear worsening from CIS to the PMS group) but also spreads from the ONH towards the fovea (Fig. 3A, C and E). In the early stages (CIS), RNFL atrophy is primarily observed near the ONH. In the relapsing-remitting phase (RMS), the atrophy expands to involve regions near the ONH as well as the superior and inferior perifoveal zones. Finally, in the early progressive phase (PMS), the RNFL atrophy occupies the entire nasal macula, spanning the region between the ONH and the fovea. Interestingly, these patterns of RNFL atrophy correspond to the vulnerability of specific retinal fibre bundles, namely, the papillomacular bundle (PMB) and the upper/lower arcuate bundles **(**Supplementary Fig. 2**)**, which notably consist of small-diameter axons.^15^ The selective vulnerability of small fibres in multiple sclerosis aligns with neuropathological studies in both the optic nerve and other CNS areas.^4,42^ Based on the consistent pattern of RNFL atrophy observed and the fibre projections, we speculate that this atrophy pattern suggests retrograde degeneration from the axons of the RGCs towards their cell bodies. Another possible explanation is that RNFL atrophy detected with VBM-OCT captured subclinical optic nerve involvement due to subtle inflammatory activity at a level not sufficient to induce symptoms or nerve conduction changes and in some ways associated to inflammatory activities present in other CNS areas.

On the other hand, GCIPL thinning also increases in severity with disease duration, with PMS displaying almost twice the GCIPL thinning seen in CIS and RMS. However, in contrast to the RNFL patterns described above, GCIPL atrophy remains confined to C-shaped areas in the nasal macula across all the disease subtypes (Fig. 4A, B, C and E). The discrepancy between RNFL and GCIPL atrophy patterns suggests that GCIPL atrophy is not solely due to retrograde degeneration from axonal damage. Prior literature has suggested that the retina itself may be a primary target involved in the pathophysiological process of multiple sclerosis, leading neuronal damage detectable since the disease onset, and that there may exist a selective vulnerability of certain types of RGCs.^4,43^ This observation aligns with our previous work, demonstrating that GCIPL atrophy is associated with disease burden in early multiple sclerosis and CIS, independent of prior optic neuritis or subclinical evidences of it.^19^ Moreover, it is noteworthy that the selective vulnerability may also target RGC axons rather than the cell bodies, as previous studies have shown that axons with smaller diameters are susceptible to injury in multiple sclerosis.^44^

Overall, VBM-OCT offers an opportunity to monitor the two mechanisms and investigate how they interact, potentially leading to an acceleration of neurodegeneration propagation (Fig. 4G and H). Furthermore, the atrophy detected with VBM-OCT was not uniformly distributed across the entire macula but rather clustered in specific areas. This crucial topographical information was previously overlooked when using standard parameters, thus limiting the sensitivity of OCT in capturing early neurodegeneration.

Recently Lublin *et al*.^45^ analysed the NO.MS dataset with over 27 000 PwMS and confirmed that progression independent from relapse activity (PIRA) occurs across all disease phenotypes and is the main driver for disease worsening. Another study using the MS-EPIC dataset emphasized that continuous brain atrophy was found even in patients without relapses or clinical worsening.^46^ Clinical and neuropathological evidence highlights the importance of chronic inflammation and disease progression, as diffuse, continuous inflammation is present in all multiple sclerosis phenotypes and seems to play a crucial role in progressive neurodegeneration.^47,48^ Therefore, instead of the current clinically based definition of progression, there is a need to shift to biomarker-based, biologically informed definitions for more effective treatment decisions.^47^ VBM-OCT has the potential to be developed into biomarkers capable of quantifying subclinical neurodegeneration on the individual level. The possibility of applying VBM to individual-level analysis as a biomarker has been demonstrated in early Huntington’s disease with MRI.^49^ Future work should focus on realizing VBM-OCT at the individual level and evaluating its sensitivity and specificity as a biomarker for neurodegeneration. Additionally, our findings indicate that both age and EDSS scores are correlated with RNFL and GCIPL atrophy. Therefore, longitudinal follow-ups with VBM-OCT can also provide insights into how aging interacts with neurodegeneration and its relationship to clinical disability.

### VBM-OCT maps show co-localization of INL thickening with RGC damage

The INL demonstrates various dynamic abnormalities in multiple sclerosis, including microcystic macular oedema (MME), transient thickening in association with inflammatory disease activity, and atrophy in late, progressive disease.^50,51^ In our study, we observed widespread clusters of INL thickening in the parafoveal zone in all disease groups, mainly alongside GCIPL atrophy. Since none of the patients exhibited MME and we screened for possible retinopathies that may affect INL thickness (in adherence to the OSCAR-IB guideline for OCT), we believe that the INL thickening observed in the present study is unlikely due to MME. The intra-group overlapped maps demonstrated that INL thickening is not simply a result of physical attraction from the RGC loss. Rather, the clustered nature of the INL thickening supports the hypothesis that it reflects a reaction of Müller cells to the loss of RGCs, rather than widespread glymphatic overload.^19^ However, given that the reliability of INL is only moderate, a larger sample size is needed to confirm this hypothesis. INL does not present progressive thinning in the early stages of the disease but eventually becomes affected by the pathology of multiple sclerosis in the progressive stages of the disease.^52-54^ Our study focused on the early stages of the disease, with even the PMS group having a disease duration of only 8.8 years. However, it is possible that the presence of INL thinning due to neurodegenerative mechanisms counteracts the INL thickening discussed here, leading to an underestimation of the degree of thickening detected with VBM-OCT.

The current study has several limitations that should be considered. Firstly, it is an exploratory study with a relatively small sample size, which may limit the generalizability of the findings. Additionally, the effects of DMTs, the time lapse between the first attack and OCT acquisition in patients experiencing their first neurological episodes and the effects of neurodegeneration in the fellow eye in the case of ON were not examined due to the sample size constraints. Moreover, while people with diabetes were excluded, other comorbidities such as obesity or hypertension were not investigated. Furthermore, the reliability of the VBM-OCT was found to be higher in the nasal macula, suggesting that changes in the temporal macula may be underestimated. This should be taken into account when interpreting the results. Another limitation is the cross-sectional design of the study, which makes it challenging to establish causality between the observed retinal changes and their relationship with clinical features. A larger, longitudinal study would be required to confirm the current observations and allow for a more comprehensive interpretation of the findings. Moreover, the observed retinal changes lack validation regarding their underlying mechanisms. Future studies should incorporate biological, neurophysiological, neuroradiological, and post-mortem pathological analyses to better understand the biological basis of the retinal alterations detected with VBM-OCT. Last, it is important to note that this study was conducted at a single site, and all OCT images were acquired from one scanner. Therefore, future multicentre studies should be performed to evaluate the reproducibility of the current analytical pipeline across different centres and scanners.

To conclude, our study highlights the potential of VBM-OCT as a sensitive tool for detecting and monitoring neurodegeneration in PwMS. Our results showed that GCIPL atrophy can be detected from the onset of the disease, even in patients who experienced their first demyelinating attack and showed no subclinical optic nerve involvement. Additionally, RNFL atrophy at entry can predict a second attack in this patient cohort. Furthermore, our findings reveal the bidirectional neurodegeneration of RNFL and GCIPL atrophy, which spreads as the disease progresses, and can be monitored with VBM-OCT. The ability of VBM-OCT to perform statistical comparisons and preserve topographical information provides valuable insights into the pathogenesis of multiple sclerosis and offers a novel endpoint in clinical trials targeting neuroprotection. VBM-OCT has the potential to serve as a biomarker reflecting the status of neurodegeneration at the individual patient level, facilitating personalized treatment decisions and monitoring disease progression.

## Supplementary Material

fcad249_Supplementary_Data

## Data Availability

The data are not publicly available due to patient confidentiality. The data presented in this study are available upon reasonable request to the corresponding author.
