# Correction to: Optical coherence tomography with voxel-based morphometry: a new tool to unveil focal retinal neurodegeneration in multiple sclerosis

**DOI:** 10.1093/braincomms/fcaf269

**Published:** 2025-07-28

This is a correction to: Su-Chun Huang, Marco Pisa, Simone Guerrieri, Gloria Dalla Costa, Giancarlo Comi, Letizia Leocani, Optical coherence tomography with voxel-based morphometry: a new tool to unveil focal retinal neurodegeneration in multiple sclerosis, *Brain Communications*, Volume 6, Issue 1, 2024, fcad249, https://doi.org/10.1093/braincomms/fcad249

In the originally published version of this paper there was an error in affiliation 1. This was given as “Experimental Neurophysiology Unit, Institute of Experimental Neurology-INSPE, San Raffaele Scientific Institute, Milan 20132, Italy” but should have been given as “Experimental Neurophysiology Unit, Institute of Experimental Neurology-INSPE, IRCCS San Raffaele Scientific Institute, Milan 20132, Italy”.

These details have been corrected only in this correction notice to preserve the published version of record.

