# Supplementary material for: Optical coherence tomography with voxel-based morphometry: a new tool to unveil focal retinal neurodegeneration in multiple sclerosis
Source: Brain Commun. 2023 Sep 28;6(1):fcad249. doi: 10.1093/braincomms/fcad249 (PMC10847824; doi:10.1093/braincomms/fcad249)
Supplement: fcad249_Supplementary_Data [file fcad249_supplementary_data.pdf]

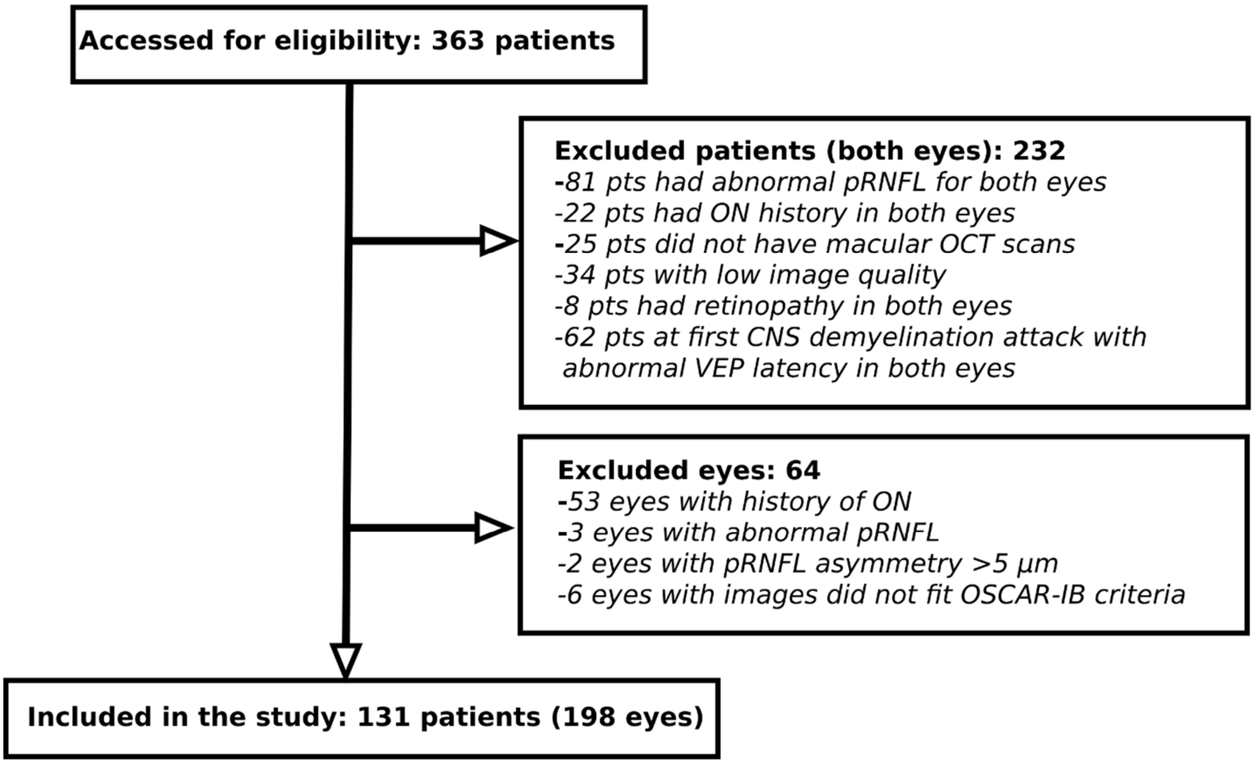

Supplementary Figure 1. Patient Inclusion Diagram. A total of 363 patients underwent VEP/OCT examinations between September 2013 and September 2018. After the screening process, 198 eyes from 131 patients met the inclusion criteria and were included in the present study.

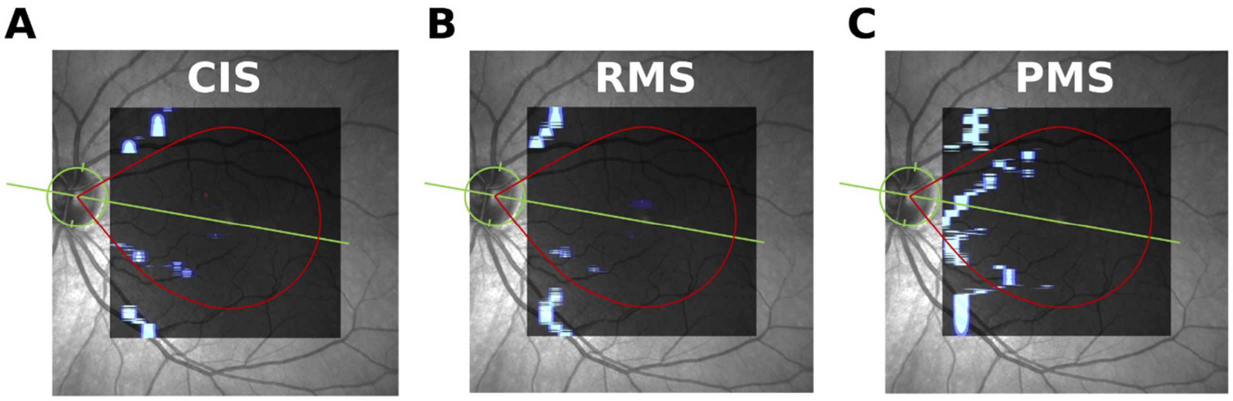

Supplementary Figure 2. Involvement of Papillo-macular bundle. To investigate the involvement of the papillo-macular bundle (PMB) in multiple sclerosis, we defined the PMB area (shown in red) based on recent literature[1]. We then superimposed this PMB area onto the RNFL atrophy map derived from Figure 3. The RNFL atrophy (blue) detected in the MS subgroups, when comparing with the HC group, was predominantly located in the arcuate bundles and the PMB. These findings highlight the selective vulnerability of the PMB region in multiple sclerosis related neurodegeneration.

[1] Leung CKS, Guo PY, Lam AKN. Retinal Nerve Fiber Layer Optical Texture Analysis: Involvement of the Papillomacular Bundle and Papillofoveal Bundle in Early Glaucoma. Ophthalmology. Sep 2022;129(9):1043-1055. doi:10.1016/j.ophtha.2022.04.012
